# Cure for Burns

**Published:** 1891-06

**Authors:** 


### CURE FOR BURNS.

Put the part instantly in cold water, or cover it with moistened baking powder, and then with a wet cloth. When the skin is destroyed the point to be attained is to exclude the air ; do this by covering the burn with sweet oil, vaseline, linseed oil, cream, carron oil, lard, or with flour spread thickly on a linen cloth or on a cotton batting. An excellent covering for burnt surfaces is made by mixing common whiting (used in kitchens for polishing purposes) with sweet oil, olive, cottonseed or other oil, or even water, into a thick paste. With this the burn is carefully covered by means of a feather, taking care not to break the blister, then the whole part is covered with cotton cloth and kept clean and moist. Burns of large size are always dangerous, often resulting in death, and should receive the careful attention of a skilled physician.

